# Insecticidal Effect of Four Insecticides for the Control of Different Populations of Three Stored-Product Beetle Species

**DOI:** 10.3390/insects13040325

**Published:** 2022-03-25

**Authors:** Georgia V. Baliota, Evagelia Lampiri, Evanthia N. Batzogianni, Christos G. Athanassiou

**Affiliations:** 1 Laboratory of Entomology and Agricultural Zoology, Department of Agriculture, Crop Production and Rural Environment, University of Thessaly, Phytokou Street, Nea Ionia, 38446 Magnesia, Greece; elampiri@agr.uth.gr (E.L.); athanassiou@agr.uth.gr (C.G.A.); 2 THESGI Farmers’ Cooperative of Thessaly, 3rd klm Larissa-Volos, 41336 Larissa, Greece; ebatzogianni@thesgi.gr

**Keywords:** stored-product insects, grain protectants, phosphine, tolerance, insect strains

## Abstract

**Simple Summary:**

Insecticides are currently the most effective method to control stored product insect pests worldwide. However, insecticide resistance poses a continuous threat to the viability of these management tools and thus, on food availability. Since there is very limited information available on the existence of resistant/tolerant insect populations in Greece, the objective of our study was to investigate the tolerance status of insect populations sampled from Greek warehouses and silos to a wide range of insecticides. According to our data, all field-collected insect populations indicated different patterns of tolerance, suggesting the occurrence of possible resistance to widely used insecticides. Our findings can be used for the reduction of the cases of control failures by revising the current pest management practices followed by Greek farmers and operators in stored product protection.

**Abstract:**

The protection of stored products from insect pests is mainly based on suppressive methods by using contact and gaseous insecticides, globally. Following their continuous and improper use, insecticide resistance has been observed in several major insect species and pose a continuous threat to the sustainability of a wide range of active ingredients that are currently in use in stored product protection. In the present work, on-site samplings of insect populations were carried out in local warehouses containing different types of cereals. The collected insects, *Rhyzopertha dominica* (F.) (Coleoptera: Bostrychidae), *Sitophilus zeamais* Motschulsky (Coleoptera: Curculionidae) and *Cryptolestes ferrugineus* (Stephens) (Coleoptera: Laemophloeidae), were reared under laboratory conditions to determine tolerance/resistance to widely used insecticides, using different diagnostic protocols. Laboratory populations of the same species were also examined for comparative purposes. Adult knock down and mortality of all populations indicated different patterns of tolerance to phosphine, deltamethrin, cypermethrin, and pirimiphos-methyl. In many cases, the recommended label doses were not able to completely control some of these populations, regardless of their origin, i.e., field-collected or laboratory. The results of the present work underline the importance of population on the efficacy of insecticides that are currently in use in stored product protection.

## 1. Introduction

Insects can cause significant quantitative losses and qualitative degradations in stored agricultural products. Thus, food protection from such pests is of great importance in order to prevent postharvest losses and secure food availability. In principle, insects have had to constantly cope with an environment containing target-specific hazards and stress conditions they had never previously encountered [1]. In this context, stored-product insect species that have been reported as significant pests from ancient times are still the main focus of pest management worldwide [2], and as such, they constitute a continuous threat on food up to date, due to their rapid genetic or behavioral adaptations, such as the development of resistance to insecticides.

The first case of insecticide resistance was reported over a century ago [3]. However, resistant pest species became a serious threat only after the introduction of synthetic organic insecticides in the 1940s. Not surprisingly, research over organophosphorus (OP) compounds, such as pirimiphos-methyl, along with the synthetic pyrethroids, such as deltamethrin and cypermethrin, as well as the fumigant phosphine, attracted most of the scientific interest for resistance studies due to their extensive utilization in stored product protection [4,5,6]. Pirimiphos-methyl was first introduced as a replacement after malathion’s control failures [7] and has been used extensively ever since as a broad-spectrum contact insecticide [8,9] for grain or structural applications [10,11,12]. Deltamethrin and cypermethrin, also broad-spectrum contact insecticides, are well-known for use against insects of public health importance (mosquitos, cockroaches etc.) [13,14,15] due to their relatively low toxicity to mammals and humans [16]. Furthermore, they are commonly used to replace older or banned insecticides in stored product protection [17,18]. Phosphine is currently the most common fumigant in stored product protection globally [19]. Its wide industrial use lays in its ability to be easily applied and penetrate well into commodities, killing all life stages of insects and, although it is a highly poisonous liquefied gas, it can be removed rapidly by aeration after application, leaving no dangerous residues on the food [19,20]. However, despite phosphine’s proved efficacy in a wide range of application scenarios, control failures are commonly observed, either by improper fumigation practices (uneven gas distribution, poor sealing, low concentrations, etc.) [21,22] or by the occurrence of resistant insect populations [23,24].

Consequently, insecticide resistance is a frequent shortcoming linked to the intensive use of these compounds in many areas. A plethora of reports have been published regarding resistant populations of major stored product pests. For example, resistant populations of the maize weevil, *Sitophilus zeamais* Motschulsky (Coleoptera: Curculionidae), the granary weevil, *Sitophilus granarius* (L.) (Coleoptera: Curculionidae), the saw-toothed grain beetle, *Oryzaephilus surinamensis* (L.) (Coleoptera: Silvanidae), the red flour beetle, *Tribolium castaneum* (Herbst) (Coleoptera: Tenebrionidae), and the lesser grain borer, *Rhyzopertha dominica* (F.) (Coleoptera: Bostrychidae) to pirimiphos-methyl have been reported in Turkey [25], Australia [26], Africa [27], Europe [28], Mexico [29], and Brazil [30]. Additionally, resistant populations to deltamethrin and cypermethrin, which also have been initially utilized to alleviate resistance to OP compounds, have been recorded in large geographical areas [9,29,31,32,33]. Resistance to phosphine has been recorded and quantified in Europe [34], USA [35], China [36], India [37], Brazil [38], and Australia [24], and is considered an issue of major importance for the continued effectiveness of this fumigant in pest management strategies [19,35,39,40]. Nevertheless, resistance parameters are “quantified” as a comparative efficacy of the insecticide that is to be tested among different populations of the same species, usually by comparing susceptible populations that are typically kept at the laboratory for many years with the ones that are under investigation. Still, this effort may provide useful results when one target active ingredient is tested, while screenings with a wide range of active ingredients may provide dissimilar results when the same laboratory populations are used [34,41].

The rapid spread of stored-product insect populations through international trade constitutes the essential need for further evaluation of this phenomenon with a targeted approach in terms of the insecticides that are currently in use locally at the industrial level, with suggestions for corrective actions. In Greece, data about the occurrence of resistant populations are scarce, although the long-term storage of grains is a common practice on local warehouses and silos. Therefore, the objective of our study was to detect and quantify tolerance/resistance to pirimiphos-methyl, deltamethrin, cypermethrin, and phosphine, which are registered for grain protection in Greece, in on-site sampled (field) populations of *R. dominica*, *S. zeamais*, and the rusty grain beetle, *Cryptolestes ferrugineus* (Stephens) (Coleoptera: Laemophloeidae) infesting stored cereals in the region of Thessaly, in comparison with standard laboratory populations.

## 2. Materials and Methods

### 2.1. Insect Populations

Field populations were sampled from storage facilities across the region of Thessaly, which is the major grain-producing geographical zone in Greece. To obtain the field populations, samples of infested maize, barley, and hard wheat were collected from the facilities on spring 2021, transferred to the laboratory, and screened individually for live adults, which were then identified up to the species level using the key of Gorham [42]. In total, three field populations of *R. dominica*, *S. zeamais*, and *C. ferrugineus* were established, each of which was reared individually by species and grain sample. To rear the insects, approximately 50 live adults were put into culturing glass jars filled with 500 g of cracked soft wheat kernels for *R. dominica* and *S. zeamais* or wheat bran with 5% soft wheat kernels for *C. ferrugineus*, with different series of jars for each species and population. The standard laboratory reference populations of the same species were also examined for comparative purposes. These laboratory populations have been maintained in the Laboratory of Entomology and Agricultural Zoology (LEAZ), Department of Agriculture, Crop Production and Rural Environment, University of Thessaly, for more than 15 years with no exposure to insecticides. The same rearing procedure and media for the field populations were also used for the laboratory populations of the same species. All insect cultures (field and laboratory) were kept in incubator chambers set at 25 °C and 65% relative humidity (RH) under continuous darkness, for a period of 2 weeks. Then, all the initial adults were removed and the rearings were placed back to the incubators to record F1 adult emergence. The emerged adults, which were of mixed sex and 7–21-d old, were used in the tests.

### 2.2. Contact Insecticides

The commercial formulations of the active ingredients (a.i.) deltamethrin (Seguro 2,5 EC, Agrotechnica, Thessaloniki, Greece), cypermethrin (Farmathrin 10ES, Farma-Chem SA, Thessaloniki, Greece), and pirimiphos-methyl (Actellic 50 EC, Syngenta Hellas, Attiki, Greece) were used in the experiments. These insecticides were chosen according to their wide use for disinfestation of stored product facilities worldwide [4,9,33]. The label doses, i.e., 1 ppm of deltamethrin and cypermethrin and 4 ppm of pirimiphos-methyl, along with a ten-fold dose, i.e., 10 ppm of deltamethrin and cypermethrin and 40 ppm of pirimiphos-methyl, were applied in uninfested and insecticide-free soft wheat. Insecticide spraying solutions were prepared by diluting the appropriate amounts of each insecticide in 100 mL of distilled water, i.e., 0.8, 0.2, and 1.6 mL, for the lower doses and 8, 2, and 16 mL for the higher doses of deltamethrin, cypermethrin, and pirimiphos-methyl, respectively, for spraying 1 mL of solution per 200 g of wheat. The solutions were applied in the commodity by using a specialized airbrush (Badger 100, Kyoto BD-183 K Grapho-tech, Japan), with different series of wheat lots for every combination of insecticide and dose rate. An additional series of wheat lots that were sprayed with water in parallel to each treatment was used as untreated control (0 ppm). Ten grams (10 g) of grain samples from each treatment and ten adults of the populations were placed inside plastic cylindrical vials (Rotilabo^®^-sample tins with snap-on lid, 3 cm in diameter, 8 cm in height, Carl Roth Gmbh & Co., Kg, Karlsruhe, Germany), with different sets of vials for each insect species × population × insecticide × dose rate. All vials had small holes in the lid for proper ventilation. Moreover, the internal “neck” of the vials was covered with Fluon (polytetrafluoroethylene, Northern Products, Woonsocket, RI) to prevent insects from escaping. The entire experiment was repeated two times (jars) with each containing three subreplicates (vials), for each combination treatment. All vials were maintained in incubators set at 25 °C, 55% RH, and continuous darkness. Insect mortality was evaluated after 3, 9, and 14 days of exposure to the treated commodity.

### 2.3. Phosphine

The standard Phosphine Tolerance kit (PTT) (Detia Degesch GmbH, Laudenbach, Germany) was used for the evaluation of tolerance to phosphine, according to the methodology described by Steuerwald et al. [43] and later modified by Athanassiou et al. [44]. Based on this method, 20 adults of each species and population were placed separately in a plastic kit syringe with 100 mL capacity. Phosphine was produced by adding 2 kit tables to 50 mL water within a flexible gas-tight plastic canister of 5 L in capacity. Concentration of the gas produced inside the canister was determined by using glass tubes [43,44]. Then, a specific quantity was removed from the canister with the syringe in order to achieve a concentration of 3000 ppm. Individual insects inside the syringe were exposed for 5, 10, 15, 20, 30, 40, 60, and 90 min (min) and after each exposure interval, they were monitored and classified as active (able to walk normally) or knocked down, i.e., incapable of coordinating movement or immobilized individuals. The classification of these individuals was based on the absence of movement, not on mortality as suggested by Agrafioti et al. [34]. For each population, there were three replicates (canisters) with two sub-replicates (syringes), with new phosphine production each time. The occurrence of tolerance to phosphine between the species’ populations was examined based on the critical times recommended by Athanassiou et al. [44].

### 2.4. Statistical Analysis

For the mortality tests of the contact insecticides, the effects of the treatments on adult mortality were analyzed separately for each species using the MANOVA fit repeated-measures procedure with Wilk’s lambda test by using JMP software [45], with insect mortality at each exposure interval as the response variable and population, insecticide, and dose rate as the main effects. A one-way ANOVA was performed using the same software, with mortality as the response variable and dose as the main effects, within each population, insecticide, and exposure period. Means were separated by the Tukey–Kramer HSD test at 0.05 within the same exposure interval. For the PTT protocol, the data were analyzed separately for each population by using probit analysis to estimate the knock down time, i.e., KDt_50_, KDt_95_, and KDt_99_, which was based on the sum of knocked-down insects. Regression analysis for field populations was applied by using the SPSS Statistical Analysis [46]. Student’s *t*-test was used to determine differences between the populations of the same species, within the same insecticide, dose, and exposure interval.

## 3. Results

### 3.1. Contact Insecticides

**Figure 1:**
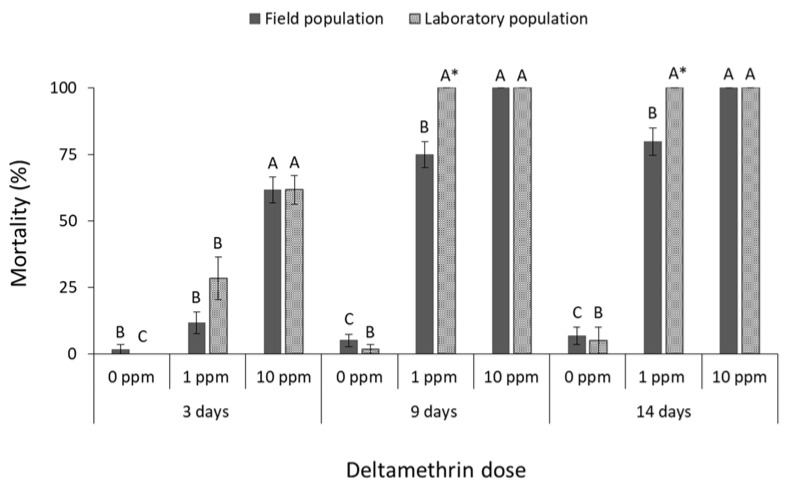
Mean (% ±SE) adult mortality of field and laboratory populations of *R. dominica* after 3, 9, and 14 days of exposure to different concentrations of deltamethrin (within each exposure interval and population, means followed by the same letter are not significantly different; where no letters are present, no significant differences were noted; HSD test a 0.05; within each exposure interval and dose, means with asterisk (*) indicate significant differences between the populations, according to Students’ *t*-test at *p* < 0.05).

**Figure 2:**
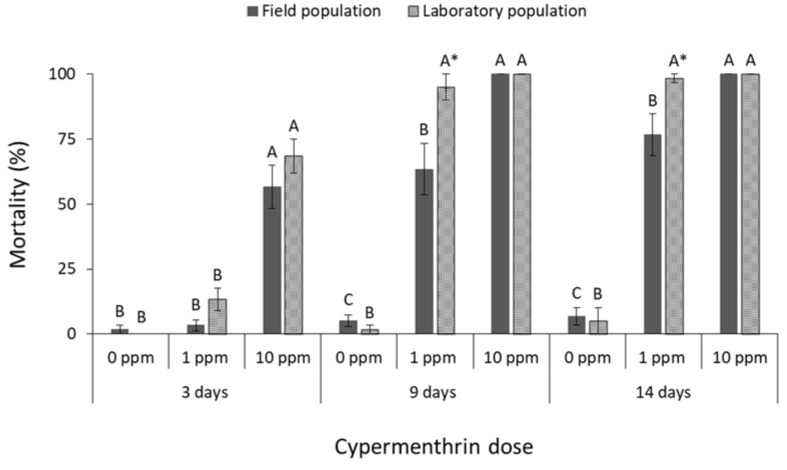
Mean (% ±SE) adult mortality of field and laboratory populations of *R. dominica* after 3, 9 and 14 days of exposure to different concentrations of cypermethrin (within each exposure interval and population, means followed by the same letter are not significantly different; where no letters are present, no significant differences were noted; HSD test a 0.05; within each exposure interval and dose, means with asterisk (*) indicate significant differences between the populations, according to Students’ *t*-test at *p* < 0.05).

**Figure 3:**
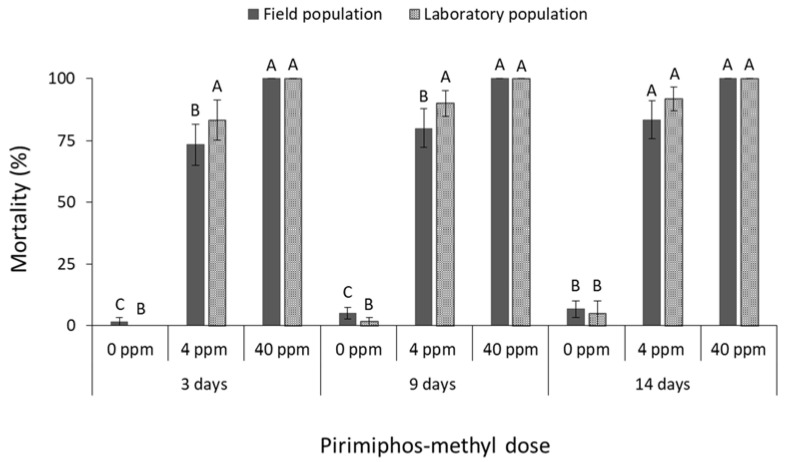
Mean (% ±SE) adult mortality of field and laboratory populations of *R. dominica* after 3, 9 and 14 days of exposure to different concentrations of pirimiphos-methyl (within each exposure interval and population, means followed by the same letter are not significantly different; where no letters are present, no significant differences were noted; HSD test a 0.05).

The mortality of all species was significantly affected by main effects and their associate interactions in most of the cases (Table 1). The mortality of *R. dominica* was significantly affected by deltamethrin doses (field population at 3 days: F = 74.4, *p* < 0.01, at 9 days: F = 242.5, *p* < 0.01, at 14 days: F = 191.8, *p* < 0.01; laboratory population at 3 days: F = 31.0, *p* < 0.01, at 9 days: F = 3481.0, *p* < 0.01, at 14 days: F = 361.0, *p* < 0.01; in all cases df = 17) (Figure 1). After 9 and 14 days, the label dose (1 ppm) was found to be significantly less effective against the field population of *R. dominica* in comparison with the corresponding laboratory population (at 1 ppm for 9 days: t = −5.0, *p* < 0.01; for 14 days: t = −3.9, *p* = 0.01; in all cases df = 11), although both populations had similar mortality at first (3 days of exposure) (Figure 1). The same pattern was observed after exposure to cypermethrin (field population at 3 days: F = 37.5, *p* < 0.01, at 9 days: F = 67.0, *p* < 0.01, at 14 days: F = 93.7, *p* < 0.01; laboratory population at 3 days: F = 65.0, *p* < 0.01, at 9 days: F = 331.3, *p* < 0.01, at 14 days: F = 319.3, *p* < 0.01; in all cases df = 17) (Figure 2), as the field population was not completely suppressed by the label dose, in contrast with the >95% mortality of the corresponding laboratory population (at 1 ppm for 9 days: t = −2.9, *p* = 0.02; for 14 days: t = −2.6, *p* = 0.04; in all cases df = 11). Response of *R. dominica* to pirimiphos-methyl was affected by doses (field population at 3 days: F = 105.0, *p* < 0.01, at 9 days: F = 115.8, *p* < 0.01, at 14 days: F = 107.9, *p* < 0.01; laboratory population at 3 days: F = 133.6, *p* < 0.01, at 9 days: F = 298.4, *p* < 0.01, at 14 days: F = 173.8, *p* < 0.01; in all cases df = 17) (Figure 3) but not by populations, since they had similar responses to all exposure doses. Nevertheless, 4 ppm could not completely control either of the populations of this species, but this was achieved at 40 ppm (Figure 3).

**Figure 4:**
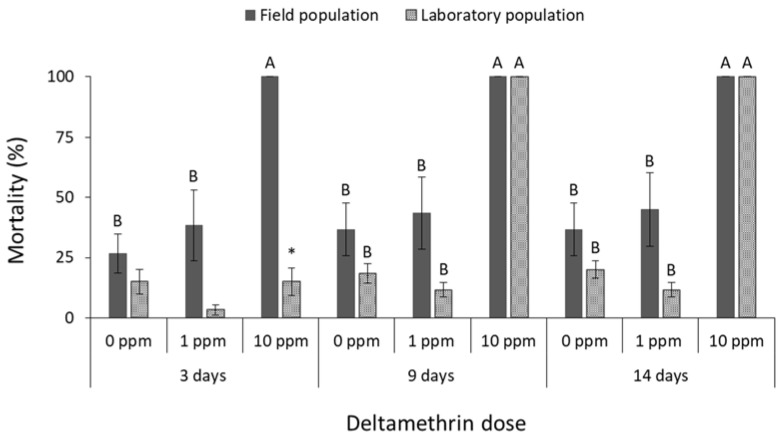
Mean (% ±SE) adult mortality of field and laboratory populations of *S. zeamais* after 3, 9 and 14 days of exposure to different concentrations of deltamethrin (within each exposure interval and population, means followed by the same letter are not significantly different; where no letters are present, no significant differences were noted; HSD test a 0.05; within each exposure interval and dose, means with asterisk (*) indicate significant differences between the populations, according to Students’ *t*-test at *p* < 0.05).

**Figure 5:**
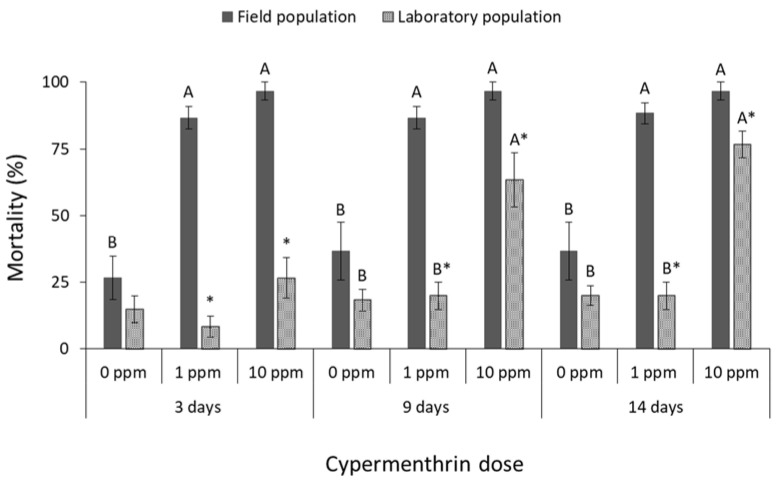
Mean (% ±SE) adult mortality of field and laboratory populations of *S. zeamais* adults after 3, 9 and 14 days of exposure to different concentrations of cypermethrin (within each exposure interval and population, means followed by the same letter are not significantly different; where no letters are present, no significant differences were noted; HSD test a 0.05; within each exposure interval and dose, means with asterisk (*) indicate significant differences between the populations, according to Students’ *t*-test at *p* < 0.05).

**Figure 6:**
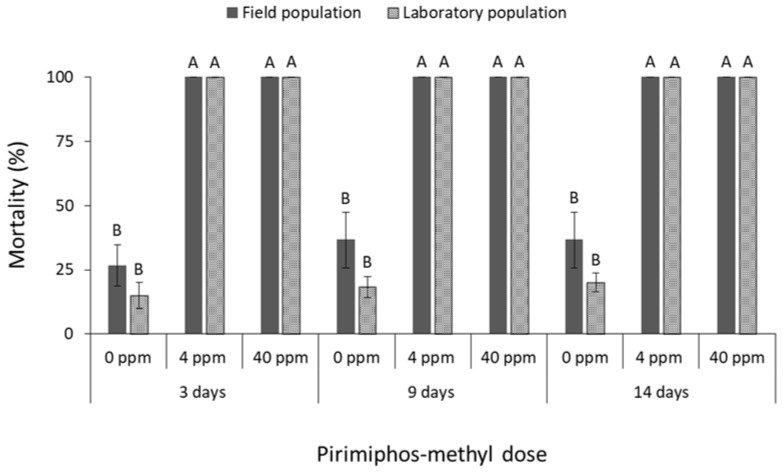
Mean (% ±SE) adult mortality of field and laboratory populations of *S. zeamais* after 3, 9 and 14 days of exposure to different concentrations of pirimiphos-methyl (within each exposure interval and population, means followed by the same letter are not significantly different; where no letters are present, no significant differences were noted; HSD test a 0.05).

The mortality of *S. zeamais* populations was significantly affected in most of the cases by deltamethrin doses (field population at 3 days: F = 16.6, *p* < 0.01, at 9 days: F = 10.6, *p* < 0.01, at 14 days: F = 10.2, *p* < 0.01; laboratory population at 3 days: F = 2.2, *p* = 0.14, at 9 days: F = 284.0, *p* < 0.01, at 14 days: F = 313.3, *p* < 0.01; in all cases df = 17) (Figure 4), by cypermethrin doses (field population at 3 days: F = 40.1, *p* < 0.01, at 9 days: F = 21.1, *p* < 0.01, at 14 days: F = 21.9, *p* < 0.01; laboratory population at 3 days: F = 2.6, *p* = 0.10, at 9 days: F = 13.3, *p* < 0.01, at 14 days: F = 49.8, *p* < 0.01; in all cases df = 17) (Figure 5), and by pirimiphos methyl doses (field population at 3 days: F = 83.5, *p* < 0.01, at 9 days: F = 34.1, *p* < 0.01, at 14 days: F = 34.1, *p* < 0.01; laboratory population at 3 days: F = 289.0, *p* < 0.01, at 9 days: F = 414.0, *p* < 0.01, at 14 days: F = 480.0, *p* < 0.01; in all cases df = 17) (Figure 6). *Sitophilus zeamais* was found to be tolerant to the label dose of deltamethrin (Figure 4), as the mortality of both populations did not exceed 45% until the end of the exposure intervals tested. Moreover, no significant differences were observed between the two populations, although the field *S. zeamais* population exhibited higher mortality rates in comparison with the laboratory population (Figure 4). Complete control was achieved for both populations exposed beyond 9 days to 10 ppm of deltamethrin, but only for the field population at the first 3 days of exposure (at 10 ppm for 3 days: t = 15.1, *p* < 0.01; in all cases df = 11). Cypermethrin was found to be the least effective insecticide against *S. zeamais* (Figure 5). Even the tenfold increase of the cypermethrin label dose had no significant effect on the mortality rates of the field population, for any of the exposure intervals examined (Figure 5). Furthermore, the laboratory *S. zeamais* population was found to be significantly more tolerant than the corresponding field population in all examined doses and exposure intervals (at 1 ppm for 3 days: t = 13.5, *p* < 0.01; for 9 days: t = 10.0, *p* < 0.01; for 14 days: t = 10.5, *p* < 0.01. At 10 ppm for 3 days: t = 8.4, *p* < 0.01; for 9 days: t = 3.1, *p* = 0.02; for 14 days: t = 3.4, *p* < 0.01; in all cases df = 11) (Figure 5). Finally, in the case of wheat treated with pirimiphos-methyl, 100% adult mortality was achieved for both *S. zeamais* populations at the 3-day exposure interval (Figure 6).

**Figure 7:**
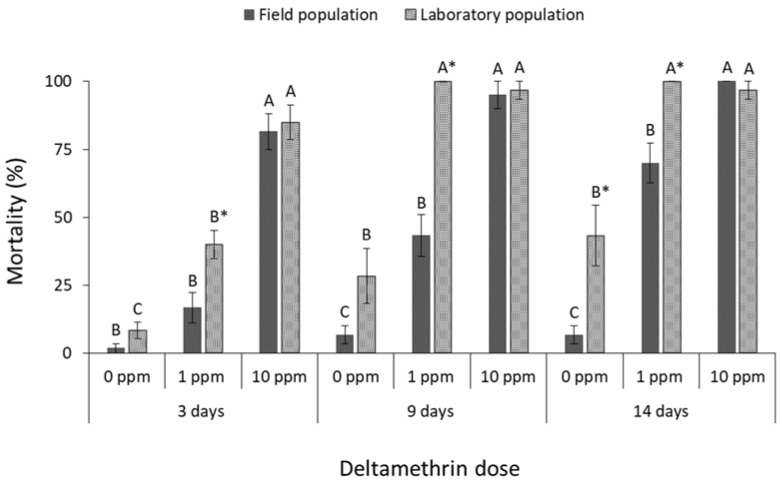
Mean (% ±SE) adult mortality of field and laboratory populations of *C. ferrugineus* after 3, 9 and 14 days of exposure to different concentrations of deltamethrin (within each exposure interval and population, means followed by the same letter are not significantly different; where no letters are present, no significant differences were noted; HSD test a 0.05; within each exposure interval and dose, means with asterisk (*) indicate significant differences between the populations, according to Students’ *t*-test at *p* < 0.05).

**Figure 8:**
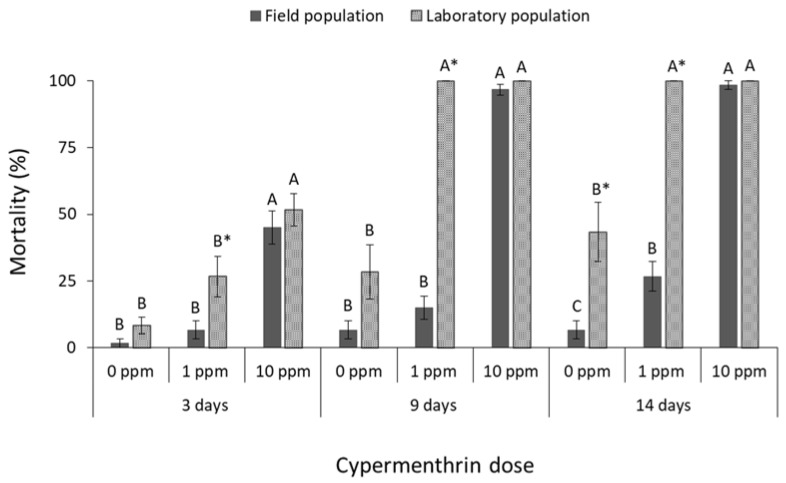
Mean (% ±SE) adult mortality of field and laboratory populations of *C. ferrugineus* after 3, 9 and 14 days of exposure to different concentrations of cypermethrin (within each exposure interval and population, means followed by the same letter are not significantly different; where no letters are present, no significant differences were noted; HSD test a 0.05; within each exposure interval and dose, means with asterisk (*) indicate significant differences between the populations, according to Students’ *t*-test at *p* < 0.05).

**Figure 9:**
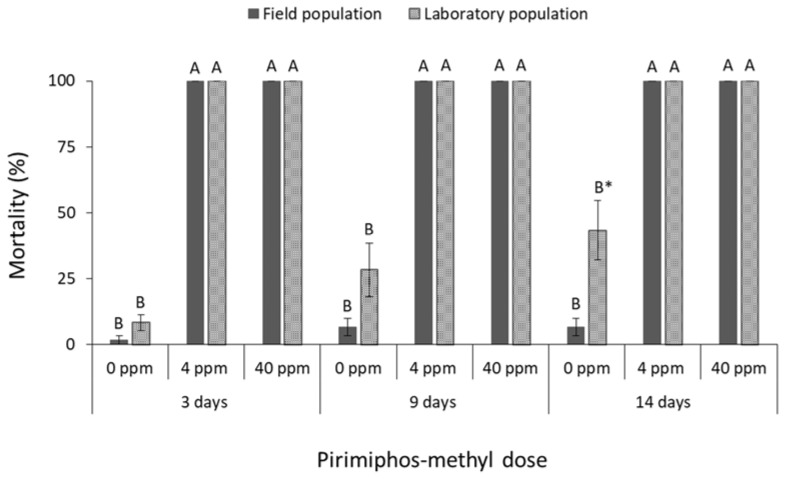
Mean (% ±SE) adult mortality of field and laboratory populations of *C. ferrugineus* after 3, 9 and 14 days of exposure to different concentrations of pirimiphos-methyl (within each exposure interval and population, means followed by the same letter are not significantly different; where no letters are present, no significant differences were noted; HSD test a 0.05; within each exposure interval and dose, means with asterisk (*) indicate significant differences between the populations, according to Students’ *t*-test at *p* < 0.05).

Significant differences were noted in adult mortality of both *C. ferrugineus* populations among deltamethrin doses (field population at 3 days: F = 70.8, *p* < 0.01, at 9 days: F = 62.9, *p* < 0.01, at 14 days: F = 105.7, *p* < 0.01; laboratory population at 3 days: F = 59.8, *p* < 0.01, at 9 days: F = 43.1, *p* < 0.01, at 14 days: F = 22.4, *p* < 0.01; in all cases df = 17) (Figure 7), among cypermethrin doses (field population at 3 days: F = 32.3, *p* < 0.01, at 9 days: F = 218.9, *p* < 0.01, at 14 days: F = 154.9, *p* < 0.01; laboratory population at 3 days: F = 13.7 *p* < 0.01, at 9 days: F = 50.0, *p* < 0.01, at 14 days: F = 25.8, *p* < 0.01; in all cases df = 17) (Figure 8), and among pirimiphos-methyl doses (field population at 3 days: F = 3481.0, *p* < 0.01, at 9 days: F = 784.0, *p* < 0.01, at 14 days: F = 784.0, *p* < 0.01; laboratory population at 3 days: F = 889.7, *p* < 0.01, at 9 days: F = 49.9, *p* < 0.01, at 14 days: F = 25.8, *p* < 0.01; in all cases df = 17) (Figure 9). The field *C. ferrugineus* population was found to be significantly less susceptible (<70% mortality) than the corresponding laboratory population to the label dose of deltamethrin, regardless of the exposure period (at 0 ppm for 14 days: t = −3.1, *p* = 0.02. At 1 ppm for 3 days: t = −3.1, *p* = 0.01; for 9 days: t = −7.5, *p* < 0.01; for 14 days: t = −4.1, *p* < 0.01; in all cases df = 11) (Figure 7). However, the increase of deltamethrin dose significantly increased the mortality of the field population in all exposure intervals, resulting to 100% mortality after 14 days. On the contrary, the laboratory *C. ferrugineus* had similar mortality patterns (>95%) at the two doses of deltamethrin after 9 and 14 days of exposure (Figure 7). The label dose of cypermethrin did not significantly affect adults of the field *C. ferrugineus* population, as more than 70% of the population was alive after 14 days but complete control was achieved at the mid-period interval for laboratory *C. ferrugineus* population exposed to the same dose (at 0 ppm for 14 days: t = −3.1, *p* = 0.02. At 1 ppm for 3 days: t = −2.4, *p* = 0.05; for 9 days: t = −19.9, *p* < 0.01; for 14 days: t = −13.1, *p* < 0.01; in all cases df = 11) (Figure 8). However, complete suppression of both *C. ferrugineus* populations was recorded after exposure to the higher cypermethrin dose. Pirimiphos-methyl caused 100% mortality on adults of *C. ferrugineus*, regardless of the population, dose and exposure interval (at 0 ppm for 14 days: t = −3.1, *p* = 0.02; in all cases df = 11) (Figure 9).

### 3.2. Phosphine

The immobilization (knock down) rate of all field populations examined with probit analysis was relatively low, with the exception of *R. dominica* (Table 2). In general, most values did not fit the data well, since *p* values were <0.01 (Table 2). Overall, the highest KDt_99_ values among the species examined were observed for the field *C. ferrugineus* population, corresponding to 198.1 min (approximately 3.3 h), while the lowest for the field *R. dominica* population, corresponding to 15.4 min (Table 2).

**Figure 10:**
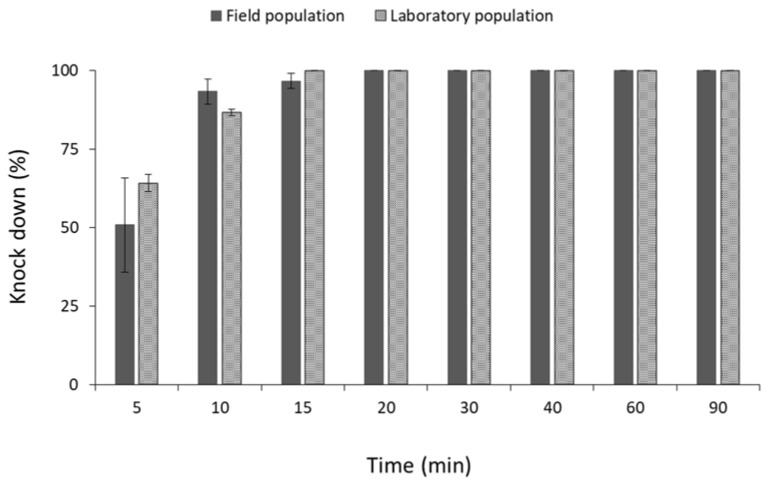
Mean (% ±SE) of knocked-down adults of field and laboratory populations of *R. dominica*, after exposure to phosphine at 3000 ppm for different observation intervals (in min).

**Figure 11:**
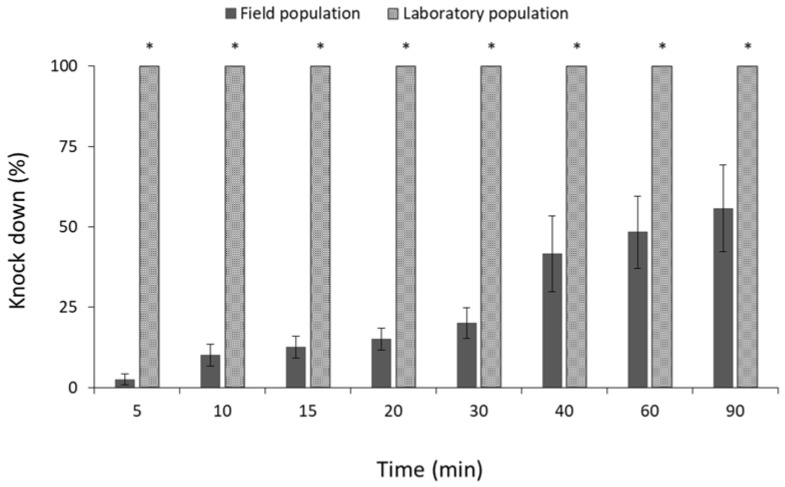
Mean (% ±SE) of knocked-down adults of field and laboratory populations of *S. zeamais*, after exposure to phosphine at 3000 ppm for different observation intervals (in min). Within each exposure interval, means with asterisk (*) indicate significant differences between the populations, according to Students’ *t*-test at *p* < 0.05.

**Figure 12:**
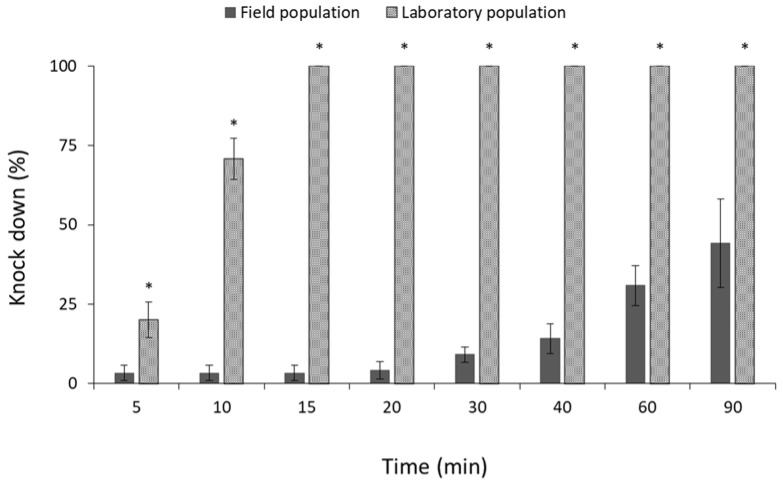
Mean (% ±SE) of knocked-down adults of field and laboratory populations of *C. ferrugineus*, after exposure to phosphine at 3000 ppm for different observation intervals (in min). Within each exposure interval, means with asterisk (*) indicate significant differences between the populations, according to Students’ *t*-test at *p* < 0.05.

Similar percentages of knocked-down adults were observed between the field and laboratory population of *R. dominica* (Figure 10). Moreover, all individuals of the same species were knocked down after 15 min of exposure, suggesting that both populations are susceptible to phosphine (Figure 10). On the contrary, significant differences were observed between the field and laboratory populations of *S. zeamais* (for 5 min: t = −57.1, *p* < 0.01; for 10 min: t = −26.3, *p* < 0.01; for 15 min: t = −26.1, *p* < 0.01; for 20 min: t = −24.9, *p* < 0.01; for 30 min: t = −16.6, *p* < 0.01; for 40 min: t = −4.9, *p* < 0.01; for 60 min: t = −4.6, *p* < 0.01; for 90 min: t = −3.3, *p* = 0.02; in all cases df = 11.) (Figure 11) and *C. ferrugineus* (for 5 min: t = −2.7, *p* = 0.03; for 10 min: t = −9.7, *p* < 0.01; for 15 min: t = −39.1, *p* < 0.01; for 20 min: t = −35.3, *p* < 0.01; for 30 min: t = −38.1, *p* < 0.01; for 40 min: t = −18.2, *p* < 0.01; for 60 min: t = −10.8, *p* < 0.01; for 90 min: t = −4.0, *p* = 0.01; in all cases df = 11.) (Figure 12). All individuals of the laboratory population of *S. zeamais* were knocked down after 5 min of exposure to phosphine, while over 45% of the field population was found to be active after the termination of the 90 min exposure (Figure 11). The same pattern was observed between the populations of *C. ferrugineus*, as 55% of the adults of the field population was capable of coordinated movement after 90 min in comparison with the 100% knocked-down laboratory population after 15 min (Figure 12). This indicates the occurrence of tolerance of the field populations of these species towards the phosphine fumigation. According to the critical times suggested by Athanassiou et al. [44], the field populations of *S. zeamais* and *C. ferrugineus* showed tolerance to phosphine.

## 4. Discussion

The sampling in this study was conducted in an attempt to highlight the occurrence of insecticide tolerance and its contribution in control failures towards the differential response of the examined stored-product beetle populations. Our field samples covered some of the most dominant pest species that generally infest stored grains in Greece [47,48]. We used two different diagnostic methods to estimate tolerance to different common insecticides: concentration-related tests for three contact insecticides and an established diagnostic protocol for the fumigant phosphine. Based on the results, we identified considerable variations in susceptibility among the field and laboratory populations within each of the four insecticides examined here.

The dose of 1 ppm of deltamethrin and cypermethrin used in our study was considered to be an effective dose for the control of the species tested here. Our overall data indicate that this dose was effective against the laboratory populations of *R. dominica* and *C. ferrugineus*. However, sufficient control of the corresponding field populations could not be achieved when the same doses and insecticides were applied, suggesting that a resistant mechanism is likely to occur. Given that pyrethroids are being applied to provide long-term protection against stored-product pests, high survival rates of insects may increase food loses by insect damage and disinfestation costs by shortening the period between insecticide applications. On the other hand, the failure of cypermethrin to control both field and laboratory population of *S. zeamais* even after increasing the dose tenfold can be considered as an indicator that the effectiveness of this insecticide may be species-dependent [49,50,51,52]. Indeed, data from Yao et al. [49] showed that different metabolic detoxification mechanisms might contribute to different insecticide susceptibility, even between closely related stored-product pest species. In this context, laboratory bioassays should be carried out when a grain protectant-based strategy is planned at a commercial scale in order to illustrate potential tolerance/resistance patterns that may render any application ineffective. Collins and Schlipalius [41] underlined the importance of the utilization of population-simplified models that are based on molecular markers and can be used to indicate gene flow processes at the population level.

Our results demonstrated that pirimiphos-methyl is effective when applied at 4 ppm. In an earlier study, Alleoni and Fereira [53] reported that all life stages of *S. zeamais* were efficiently controlled with the same commercial formulation and dose of pirimiphos methyl. Previous studies found that other *Sitophilus* species were also susceptible to pirimiphos-methyl [54,55], while this active ingredient could be used with success to control populations that were resistant to phosphine [56]. On the contrary, Rumbos et al. [54] found that 4 ppm of pirimiphos-methyl did not provide sufficient control against *R. dominica* after 14 days of exposure. Nevertheless, we did not record cross-resistance patterns between the pyrethroids tested and pirimiphos-methyl, given that the populations of *R. dominica* and *C. ferrugineus* that were tolerant to both pyrethroids were found to be susceptible to pirimiphos-methyl. Similar studies reported that deltamethrin selection demonstrated no cross-resistance to pirimiphos-methyl in *S. zeamais* [7] and *R. dominica* [57], proposing that this phenomenon is due to different genes that are involved in the resistance patterns for these two insecticides.

Considering the results of Athanassiou et al. [44] for the PTT protocol, the “critical” intervals beyond which the examined populations should be characterized as phosphine-tolerant should be between 6 and 14 min for *S. zeamais* and *C. ferrugineus*, based on KDt_99_ values for 3000 ppm. The sampled populations of *S. zeamais* and *C. ferrugineus* in this study can be clearly classified as phosphine-tolerant based on these critical intervals. However, the classification of the field *R. dominica* population towards its susceptibility to phosphine was rather vague, since the KDt_99_ values observed are similar between the field and laboratory population. This might indicate tolerance of *R. dominica* towards phosphine without previous exposure to this toxic agent, a phenomenon that has been also reported for other species [58]. As phosphine is one of the major insecticides that were in use in the facilities tested, we consider these tolerance dissimilarities between the field and laboratory populations expectable, and thus, phosphine should be rotated with some of the contact insecticides tested here.

The quick diagnostic procedure that is based on PTT can provide data for a wide range of insect populations in a short period of time, and can be definitely adopted in area-wide management strategies, as it has been illustrated by Agrafioti et al. [34]. In that study, the authors found that forty-three out of fifty-three field populations of seven stored-product pest species sampled from Greece were tolerant/resistant to phosphine. In this regard, a series of recent studies have shown that resistance of stored-product insect populations in grains that are stored in facilities in Greece is a frequent but rather neglected phenomenon. In contrast, there are disproportionally few data for these field populations to contact insecticides with different modes of action and hence, the results of the present work can be considered as the first towards this direction. The use of the residual grain protectants as an alternative solution to fumigation can be proposed as a means to mitigate tolerance/resistance to phosphine, but the continuous use of contact insecticides is equally risky on the basis of resistance development.

From a practical point of view, the absence of chemical control methods for prolonged periods is not always feasible. Still, comparing field-collected populations with laboratory populations to display variations in susceptibility may not be used for a wide range of active ingredients with a dissimilar mode of action. At the same time, it is generally common that laboratory populations are usually more susceptible to insecticides than field populations, but this may be falsely perceived as an indicator of resistance, and a comparable screening of field-collected populations may be a more reliable approach. Thus, a recent “field-collected” susceptible population could provide more realistic data in this regard, as compared with laboratory populations that are kept in controlled environments for many decades.

## Figures and Tables

**Table 1:** MANOVA parameters for mortality levels of *R. dominica*, *S. zeamais* and *C. ferrugineus* adults between or within variables (Repeated Measures ANOVA, df = 108).

Species			*R. dominica*		*S. zeamais*		*C. ferrugineus*	
Effect (Source)		df	F	*p*	F	*p*	F	*p*
Intercept		1	5494.8	<0.01	3080.2	<0.01	1763.2	<0.01
Between variabiles	Population	1	14.2	<0.01	0.5	0.47	<0.1	0.92
	Insecticide	2	85.8	<0.01	153.6	<0.01	30.2	<0.01
	Population × Insecticide	2	28.6	<0.01	8.9	<0.01	1.0	0.39
	Dose	2	1302.8	<0.01	317.6	<0.01	236.6	<0.01
	Population × Dose	2	3.8	0.02	0.9	0.40	4.3	0.02
	Insecticide × Dose	4	64.1	<0.01	45.4	<0.01	4.6	<0.01
	Population × Insecticide × Dose	4	8.6	<0.01	6.3	<0.01	5.8	<0.01
Within variables	Time	2	232.8	<0.01	214.3	<0.01	137.5	<0.01
	Time × Population	2	1.8	0.17	9.0	<0.01	0.3	0.77
	Time × Insecticide	4	6.0	<0.01	5.0	<0.01	47.2	<0.01
	Time × Population × Insecticide	4	44.0	<0.01	30.1	<0.01	1.7	0.15
	Time × Dose	4	36.9	<0.01	16.3	<0.01	18.7	<0.01
	Time × Population × Dose	4	7.3	<0.01	9.3	<0.01	0.7	0.62
	Time × Insecticide × Dose	8	12.0	<0.01	20.0	<0.01	21.1	<0.01
	Time × Population × Insecticide × Dose	8	19.2	<0.01	10.2	<0.01	1.5	0.17

**Table 2:** Probit Analysis for KDt_50_, KDt_95_ and KDt_99_ (confidence intervals) of adults after exposure to 3000 ppm concentration of phosphine for the field and laboratory populations examined, expressed as minutes to knock down, using the PTT.

Species	KDt_50_	KDt_95_	KDt_99_	Slope ± SE	X^2^	*p*
*R. dominica* (field)	4.5 (−10.1–9.2)	12.2 (6.2–15.7)	15.4 (11.4–20.0)	5.2 ± 0.4	100.9	<0.01
*S. zeamais* (field)	70.1 (43.8–91.5)	151.1 (122.2–221.2)	184.7 (146.7–282.8)	8.5 ± 0.0	178.5	<0.01
*C. ferrugineus* (field)	91.6 (78.2–115.0)	166.9 (134.8–260.3)	198.1 (156.0–322.8)	6.5 ± 0.0	119.8	<0.01
*R. dominica* (lab)	8.9 (4.2–10.4)	13.4 (11.9–16.9)	15.2 (13.4–21.4)	3.2 ± 0.1	3.1	1.0
*S. zeamais* (lab)	a	a	a	a	a	a
*C. ferrugineus* (lab)	8.8 (6.9–9.6)	12.6 (11.7–14.7)	14.2 (12.9–17.7)	3.9 ± 0.1	25.8	0.9

a Could not estimate KDt accurately.

## Data Availability

The data presented in this study are available on request from the corresponding author. The data are not publicly available due to Grand Restrictions.
